# Exchange Protein Directly Activated by cAMP Modulates Ebola Virus Uptake into Vascular Endothelial Cells

**DOI:** 10.3390/v10100563

**Published:** 2018-10-16

**Authors:** Aleksandra Drelich, Barbara Judy, Xi He, Qing Chang, Shangyi Yu, Xiang Li, Fanglin Lu, Maki Wakamiya, Vsevolod Popov, Jia Zhou, Thomas Ksiazek, Bin Gong

**Affiliations:** 1 Department of Pathology, University of Texas Medical Branch, Galveston, TX 77555, USA; aldrelic@utmb.edu (A.D.); bmjudy@utmb.edu (B.J.); xihe@utmb.edu (X.H.); qichang@utmb.edu (Q.C.); docyushangyi@163.com (S.Y.); xianli@utmb.edu (X.L.); vpopov@utmb.edu (V.P.); 2 Department of Cardiovascular Surgery, Changhai Institute of Cardiovascular Surgery, Shanghai 200433, China; lufanglin@smmu.edu.cn; 3 Department of Neuroscience and Cell Biology, University of Texas Medical Branch, Galveston, TX 77555, USA; mawakami@utmb.edu; 4 Department of Pharmacology and Toxicology, University of Texas Medical Branch, Galveston, TX 77555, USA; jizhou@utmb.edu

**Keywords:** Ebola virus, vascular endothelial cell, exchange protein directly activated by cAMP, virus uptake

## Abstract

Members of the family Filoviridae, including Ebola virus (EBOV) and Marburg virus (MARV), cause severe hemorrhagic fever in humans and nonhuman primates. Given their high lethality, a comprehensive understanding of filoviral pathogenesis is urgently needed. In the present studies, we revealed that the exchange protein directly activated by *cAMP 1* (*EPAC1*) gene deletion protects vasculature in ex vivo explants from EBOV infection. Importantly, pharmacological inhibition of EPAC1 using EPAC-specific inhibitors (ESIs) mimicked the *EPAC1* knockout phenotype in the ex vivo model. ESI treatment dramatically decreased EBOV infectivity in both ex vivo vasculature and in vitro vascular endothelial cells (ECs). Furthermore, postexposure protection of ECs against EBOV infection was conferred using ESIs. Protective efficacy of ESIs in ECs was observed also in MARV infection. Additional studies using a vesicular stomatitis virus pseudotype that expresses EBOV glycoprotein (EGP-VSV) confirmed that ESIs reduced infection in ECs. Ultrastructural studies suggested that ESIs blocked EGP-VSV internalization via inhibition of macropinocytosis. The inactivation of EPAC1 affects the early stage of viral entry after viral binding to the cell surface, but before early endosome formation, in a phosphatidylinositol-4,5-bisphosphate 3-kinase (PI3K)-dependent manner. Our study delineated a new critical role of EPAC1 during EBOV uptake into ECs.

## 1. Introduction

Members of the family Filoviridae, including Ebola virus (EBOV) and Marburg virus (MARV), cause severe hemorrhagic fever in humans and nonhuman primates, with case-fatality in humans ranging from 57% to 90% [1,2,3,4,5,6]. To date, all human outbreaks of EBOV have been traced to equatorial Africa [3]. From 2013 to 2016, the most dangerous and geographically widespread outbreak of EBOV infection in humans on record occurred in West Africa. A new EBOV outbreak was declared on 15 May 2018 in the eastern Democratic Republic of the Congo in Central Africa, a week after the western outbreak in the country was over [7]. Notably, increased travel led to the importation of EBOV and MARV into non-endemic areas around the globe [8]. Several potential vaccine candidates have moved into clinical trials, including a pseudotyped vesicular stomatitis virus that expresses EBOV glycoprotein (EGP-VSV)-based vaccine [9,10]. However, currently, neither vaccines nor postexposure treatments have been FDA-approved for combating filoviral infections [11,12,13,14]. Given their high lethality, a comprehensive understanding of filoviral pathogenesis and the development of novel mechanism-based prophylactic and therapeutic countermeasures are urgently needed.

Filoviruses seem to target both the immune system [15,16,17] and vascular endothelia [18,19,20,21], causing the most severe form of viral hemorrhagic fever (VHF). Filoviral infection initiates diffuse vascular endothelium dysfunction marked by bleeding, fluid distribution disorders, and disseminated intravascular coagulation (DIC), leading to organ failure in the latter stages of disease [17,19,22,23,24]. The vascular endothelium is a monolayer of cells lining blood vessels. Under physiological conditions, the endothelium plays a pivotal role in providing the proper hemostatic balance. Dysregulation of the barrier formed by the endothelium can increase vascular permeability and cause hypotensive shock syndrome; it is seen in many fatal diseases, including VHF cases [18,19]. However, the mechanism(s) triggering endothelium disruption during EBOV infection is still not fully understood.

Exploiting host signal pathway(s) is an effective virulence strategy employed by a number of viral hemorrhagic fever pathogens. Cyclic adenosine monophosphate (cAMP) is a common secondary ubiquitous messenger involved in several cellular processes; cAMP translates environmental signals into downstream effects at multiple cellular levels [25,26,27]. Many pathogens have developed strategies to modulate cAMP levels within host cells, including the vascular endothelium [28], by hijacking the host cell machinery for their own benefit during various stages of their life cycle, including entry, internalization, fusion, replication, and release [28,29,30,31,32]. However, it is not known if filoviruses employ similar strategies.

The effects of cAMP are transduced by two key ubiquitously expressed intracellular cAMP receptors: cAMP-dependent protein kinase A (PKA) and the recently identified exchange protein directly activated by cAMP (EPAC) [33,34,35]. EPAC activation is triggered by internal conformational changes induced by direct binding of cAMP, leading to the activation of downstream signaling effects. There are two known mammalian isoforms of EPAC, EPAC1 and EPAC2, which are encoded by independent genes that have different tissue expression patterns [33,34,35]. EPAC1 is the only isoform expressed within vascular endothelial cells (ECs) and can act independently of PKA to regulate many cellular functions induced by cAMP, including vascular barrier function and endothelial permeability, cell adhesion, cell–cell junctions, microtubule dynamics, cytokine signaling suppression, and exocytosis [36,37,38]. An in vitro study suggested that pretreatment with an EPAC-specific inhibitor (ESI), ESI09 [39], induced anti-Middle East respiratory syndrome coronavirus (MERS-CoV) and severe acute respiratory syndrome coronavirus (SARS-CoV) activities in a cell-type independent manner [40]. However, the role of EPAC during filoviral infection remains unknown.

In the present studies, we took advantage of a recently developed ex vivo vascular culture method and an in vitro mouse brain microvascular endothelial cell (BMEC) model isolated from the *EPAC1*-null mouse [41] to reveal that the *EPAC1* gene deletion protected vasculatures from infection with EBOV (see Supplemental Materials for GenBank accession number). Most importantly, pharmacological inhibition of EPAC1 using ESI09 and another ESI, NY0123 [42], mimicked the *EPAC1*-null phenotype in the ex vivo model. ESI treatment dramatically decreased EBOV infectivity in both ex vivo vasculatures and BMECs isolated from wild-type mice. Furthermore, postexposure protection of ECs against EBOV infection was conferred by pharmacological inactivation of EPAC1 using these ESIs. Employing EGP-VSV to resemble EBOV infection, we showed that ESI09 reduced EGP-VSV infection in ECs. Nevertheless, the EPAC1-specific activator (ESA) I942 [43] rendered ECs susceptible to EBOV infection after ESI09 pretreatment. Ultrastructural studies revealed that inactivation of EPAC1 blocked EGP-VSV internalization. Mechanistic studies suggested that, during the early stage of infection, activation of host phosphatidylinositol-4,5-bisphosphate 3-kinase (PI3K) can attenuate the in vitro efficacy of ESI09 against EGP-VSV infection in ECs. Our data strongly suggest that EPAC1 plays a critical role during EBOV uptake into ECs.

## 2. Materials and Methods 

### 2.1. Ethics Statement

The mouse experiments performed for this study were carried out in accordance with National Institutes of Health, United States Dept. of Agriculture, and UAMS Division of Laboratory Animal Medicine and Institutional Animal Care and Use Committee (IACUC) guidelines. The protocol supporting this study was approved by the UTMB IACUC (1506030). 

### 2.2. Ex Vivo Mouse Vasculature Assay for EBOV Infection

We established an ex vivo vascular endothelial culture model [41] for EBOV infection using aortic rings isolated from three wild-type (WT) and three *Epac1*-null mice (KO). *EPAC1* (also known as *Rapgef3*) mutant mice carrying a conditional-ready allele (B6; 129S2-*Rapgef3^tm1Geno^*/J, the Jackson Laboratory, Stock No. 018389) were obtained, and crossed with a germline Cre deletion mouse, C57BL/6-Tg(Zp3-cre)93Knw/J (The Jackson Laboratory, Stock No. 003651), to generate knockout mice. The majority of the mice used in experiments (homozygotes and wild-type controls) were C57BL/6J incipient congenic (N5) and generated by heterozygous intercrosses. Similar to published models [44,45], our *EPAC1*-null mice are viable, fertile, and without overt abnormalities. Briefly, after euthanization, aortae were first dissected from mice and then cleaned of adipose tissue, then cut into five rings per mouse aorta. A total of 30 aortic rings were cultured for 48 h in Endothelial Cell Growth Medium supplemented (Cell Applications, Atlanta, GA, USA) with 10% (vol/vol) FBS. Three rings per mouse were exposed to EBOV at 1.6 × 10^6^ plaque-forming units (pfu) in 500 µL medium in one well of a 24-well culture plate, and the other three rings from the same mouse were incubated in uninfected medium until microscopic evidence of neocellular growth was present after 1 week to confirm the viability of the cultures. At the end of the experiment, all aortic rings were immersion-fixed in 10% buffered formalin for histological analysis. After the first group of five continuous sections, tissue sections of aortic rings were collected and stored at 4 °C before being processed using immunofluorescence (IF) reagents to detect EBOV and von Willebrand factor (vWF).

### 2.3. Cell Treatments

ESI09 and PI3K-specific activator 740YP were purchased from Tocris Bioscience (Avonmouth, Bristol, United Kingdom). PKA-specific inhibitor H89 was purchased from LC Laboratories (Woburn, MA, USA). ESI NY0123 and ESA I942 were synthesized in Dr. Jia Zhou’s laboratory at UTMB. NY0123 has similar EPAC inhibitory profiles to ESI09 but is slightly more potent [46]. For in vitro studies, human umbilical vein endothelial cells (HUVECs) (Cell Applications, Atlanta, GA, USA) or human cerebral microvascular endothelial cells (hCMECs) (Applied Biological Materials, Richmond, BC, Canada) were exposed to 5 µM EIS09, NY0123, I942, or 10 µM H89 in medium for designated times prior to downstream experiments. Medium with 0.01% dimethyl sulfoxide (DMSO) (vol/vol) was used as a vehicle control. For activation of PI3K, cells were stimulated with 10 µM 740YP for 3 h. To determine the effects of ESI09, NY0123, and I942 on HUVEC viability in vitro, cytotoxicity was determined using the 3-(4,5-dimethylthiazol-2-yl)-5-(3-carboxymethoxyphenyl)-2-(4-sulfophenyl)-2H-tetrazolium (MTS) assay kit after culturing HUVECs with serial dilutions (1.25, 2.5, 5, and 10 μM) of ESI09, NY0123, and I942 separately for 24 h. Untreated HUVECs served as controls. The absorbance at 490 nm was recorded using a BioTek ELx808 plate reader (BioTek, Winooski, VT, USA). Results are presented as mean ± standard error of the mean of three independent experiments; each experiment was conducted in triplicate.

### 2.4. EBOV Infection

Cell cultures prepared as described above were inoculated with EBOV at an MOI of 0.5 for 30 min. After that time, fresh media was added, and infected cells were incubated in 5% CO_2_ in a 37 °C environment for 72 h. The cells and media from infected cells were inactivated using TriPure Isolation Reagent (Sigma Aldrich, St. Louis, MO, USA) and stored at −80 °C until RNA extraction. For immunofluorescent staining, the cells were fixed in 10% neutral-buffered formalin.

### 2.5. Binding Assay

For binding assays, DMSO- or ESI09-pretreated HUVECs were incubated with EGP-VSV at an MOI of 20 for 15 min on ice. After the incubation period, unbound viruses were removed by washing three times with ice-cold PBS. The membrane-bound viral particles were retrieved following a freeze-thaw cycle in an equal amount of 2% FBS supplemented DMEM [40]. Viral samples were stored at −80 °C for downstream assays.

### 2.6. Early Endosome Isolation and Cell Fractionation

HUVECs pretreated with ESI09 or DMSO were infected with EGP-VSV at an MOI of 200 for 1 h before thoroughly washing with PBS. Early endosome isolation and cell membrane fractionation were performed using the Minute™ Endosome Isolation and Cell Fractionation Kit (Invent Biotechnologies, Eden Prairie, MN, USA) according to the manufacturer’s instructions. The resulting fractions were resuspended in TriPure for RNA extraction or RIPA buffer for immunoblotting. The purity of the early endosome and membrane fractions was verified by immunoblotting with antibodies raised against early endosome-specific (EEA1) and membrane-specific (Na^+^, K^+^ ATPase) proteins, respectively.

### 2.7. Immunofluorescence (IF)

For dual-target IF staining of vWF and EBOV in mouse aortic rings, Ultra V Block and normal rabbit serum (DAKO) were employed for the reduction of nonspecific background before and after vWF antigen-labeling, respectively. Deparaffinized and rehydrated 5 µm sections were incubated with anti-vWF rabbit polyclonal antibody (1:100) for 2 h at 21 °C, followed by AlexaFluor 488-conjugated goat anti-rabbit IgG (1:1000) (Invitrogen, Carlsbad, CA, USA) for 30 min at 21 °C. The EBOV antigens in tissues were visualized afterward by incubation with DyLight 594 Microscale Antibody Labeling Kit labeled anti-EBOV rabbit polyclonal antibody (1:500) for 2 h at 21 °C. Nuclei were counterstained with DAPI. Fluorescent images were analyzed using an Olympus BX51 epifluorescence (Olympus Corporation, Tokyo, Japan) or Nikon Eclipse Ti confocal microscope (Nikon, Tokyo, Japan). To quantitate the viral antigen-positive cytopathic effect (CPE), cells were fixed in 10% buffered formalin. For IF staining to detect viral antigens, the fixed cell monolayers were incubated with anti-EBOV rabbit polyclonal antibody (1:500) before nuclei were counterstained with DAPI. Cells were examined and IF images were captured with an Olympus BX51 image system using a final 20× optical zoom. The number of viral antigen-positive foci (VAPF) detected in each microscopic field was manually enumerated [41] (for representative images, please see Figure S1). The results were expressed as the number of VAPF in each microscopic field. Ten microscopic fields were examined for each experiment. Data are representative of at least three experiments.

### 2.8. Statistics

Statistical significance was determined using the Student’s *t*-test or one-way analysis of variance. Results were regarded as significant if two-tailed *p* values were <0.05. All data are expressed as mean ± standard error of the mean.

## 3. Results

### 3.1. EPAC1 Gene Deletion Attenuates EBOV Infection of Ex Vivo Vasculatures and of Primary ECs In Vitro

**Figure 1:**
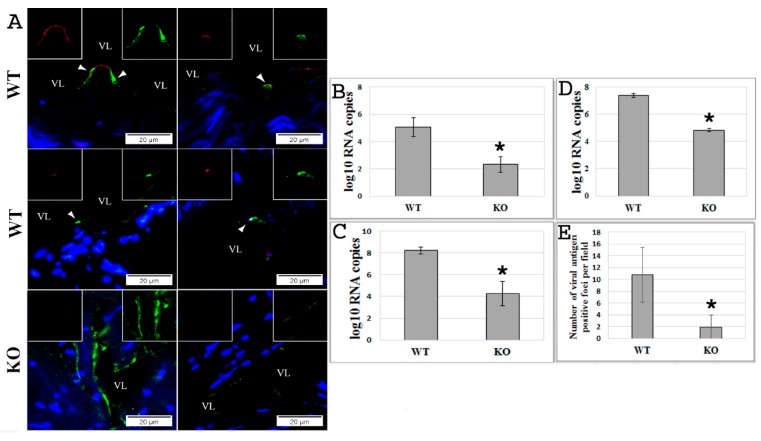
Absence of the exchange protein directly activated by *cAMP 1* (*EPAC1*) gene attenuates Ebola virus (EBOV) infection ex vivo and in vitro. (**A**) Representative dual-target immunofluorescence (IF) staining of EBOV (red) and endothelial cell (EC)-specific von Willebrand factor (vWF; green) in sections of ex vivo aortic ring vasculatures prepared from *Epac1*-null (KO) and wild-type (WT) mice 72 h postinfection (p.i.) with EBOV. Nuclei of mouse cells were counterstained with DAPI (blue). Inserts depict split signals of EBOV (red) and vWF (green) from the colocalized areas (arrow heads). Scale bars, 20 µm. VL, vascular lumen. (**B**) The number of viral RNA copies detected in the media of ex vivo aortic ring vasculatures of KO and WT mice at 72 h p.i. with EBOV. *N* = 3 for each group. (**C**,**D**) The number of viral RNA copies detected in brain microvascular endothelial cells (BMECs) (**C**) and media (**D**) at 72 h p.i. with EBOV at an MOI of 0.5. *N* = 3 for each group. (**E**) The number of viral antigen-positive foci measured using IF microscopy in the monolayers of BMECs, which were isolated from KO and WT mice, at 72 h p.i. with EBOV at an MOI of 0.5. *N* = 30 for each group. * *p* < 0.005 compared with WT groups.

The generated ex vivo vasculature model [41] using aortic rings isolated from KO or WT mice was used to examine EBOV infection. At 72 h postexposure with EBOV, it was observed that the endothelium in aortic rings from KO mice was protected from infection compared to the infected aortic rings isolated from WT mice (*p* < 0.005) (Figure 1A,B and Figure S2). This observation was further validated by an in vitro EBOV infection model of mouse BMECs prepared from KO or WT mice and infected with EBOV at an MOI of 0.5 (Figure 1C–E). The efficiency of infection was assessed by real-time PCR (qPCR) that determined the number of copies of viral RNA in the cells (Figure 1C) and media (Figure 1D), and by the formation of viral antigen-positive foci (VAPF) detected with IF staining in the infected monolayer (Figure 1E) (*p* < 0.005). The results demonstrate that deletion of the *EPAC1* gene in endothelial cells significantly reduced EBOV infection.

### 3.2. Pharmacological Inactivation of EPAC1 Protects ECs from EBOV Infection

**Figure 2:**
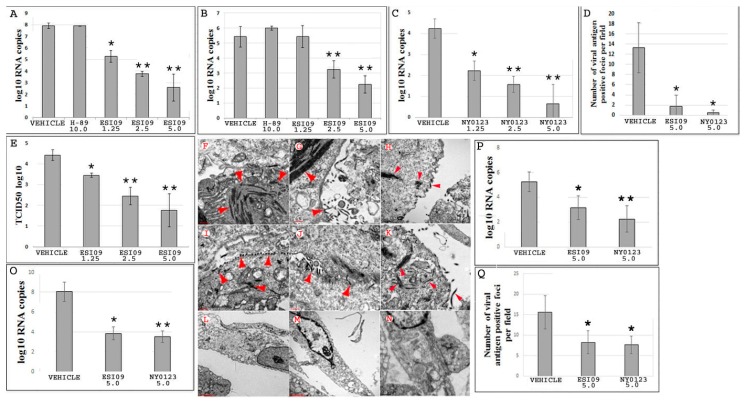
Pharmacological inactivation of EPAC1 protects human umbilical vein endothelial cells (HUVECs) from EBOV infection. (**A**,**B**) The number of viral RNA copies detected in Vehicle- (*N* = 5), ESI09- (*N* = 5), and H89-pretreated HUVECs (*N* = 3) (**A**) and media (**B**) at 72 h p.i. with EBOV at an MOI of 0.5. * *p* < 0.05, ** *p* < 0.005 compared with Vehicle groups. (**C**) The number of viral RNA copies detected in the media from Vehicle- (*N* = 3) and NY0123-pretreated (*N* = 3) HUVECs at 72 h p.i. with EBOV at an MOI of 0.5. *N* = 3 for each group. * *p* < 0.05, ** *p* < 0.005 compared with the Vehicle group. (**D**) The number of viral antigen-positive foci measured using IF microscopy in the monolayers of HUVECs at 72 h p.i. with EBOV at an MOI of 0.5. *N* = 30 for each group. * *p* < 0.05 compared with the Vehicle group. (**E**) Quantities of viral particles in media measured using the TCID50 assay, *N* = 3 for each group. * *p* < 0.05, ** *p* < 0.005 compared with the Vehicle group. (**F**–**N**) Representative electron microscopy (EM) detection of EBOV particles (arrow heads) in HUVECs pretreated with DMSO (**F**–**K**), ESI09 (**L**–**M**), and NY0123 (**N**) at 72 h p.i. with EBOV at an MOI of 0.5. EM images of 10 cells for each group were reviewed under EM. Scale bars, (**F**–**K**), 500 nm; (**L**–**N**), 2 µM. (**O**) and (**P**) HUVECs infected with EBOV at an MOI of 0.5 and treated with ESI09 and NY0123 at 5 µM 24 h later. The number of viral RNA copies detected at 72 h p.i. in the cells (**O**) and media (**P**). *N* = 3 for each group. * *p* < 0.05, ** *p* < 0.01 compared with Vehicle groups. (**Q**) The number of viral antigen-positive foci measured at 72 h p.i. using IF microscopy in the monolayers of HUVECs treated with ESI09 or NY0123 at 24 h p.i. *N* = 30 for each group. * *p* < 0.05 compared with the Vehicle group.

ESIs have been widely employed in EPAC biological research [42]. Given that EPAC1 is the only isoform expressed within the ECs [36,37,38], the potential of using EPAC pharmacological inhibition as a protective strategy for combating endothelial EBOV infection was explored. First, HUVECs were pretreated with ESI09 (5 µM), NY0123 (5 µM), or DMSO (5 µM) (Vehicle) for 24 h before challenge with EBOV for 72 h. As shown in Figure 2A–C, exposure to ESI09 significantly reduced the viral load in cells (Figure 2A) and cell media (Figure 2B), as well as in cell media after exposure to NY0123 (Figure 2C) (*p* < 0.005) at 72 h postinfection (p.i.), compared to Vehicle-treated groups. Similar inhibitory effects were confirmed by examining the formation of VAPF in the cell monolayer pretreated with either ESI09 or NY0123, which is indicative of a cytopathic effect (Figure 2D and Figure S1) (*p* < 0.05). Furthermore, viral infectivity was confirmed using the TCID50 assay to determine the infectious titer of virus in media (Figure 2E) (*p* < 0.005). Electron microscopy (EM) was also performed with HUVECs that were pretreated with either ESI09, NY0123, or DMSO and subsequently infected with EBOV to directly visualize EBOV particles (Figure 2F–N). After 72 h p.i., viral particles in ESI09- (Figure 2L,M) or NY0123-treated cells (Figure 2N) were hardly visible, whereas numerous EBOV particles at different stages of infection in the DMSO-treated group (Figure 2F–K) were found.

Both ESI09 (Figure 2A,B,E) and NY0123 (Figure 2C) pretreatments produced dose-dependent effects against EBOV infection in ECs, with the highest protection observed at a concentration of 5 µM (*p* < 0.005). It was noticeable that, even at the lowest concentration tested (1.25 µM), ESI09 or NY0123 pretreatment reduced the viral load (*p* < 0.05) (Figure 2A,C). Importantly, ESI-mediated inhibitory effects on EBOV infection were not a result of drug-mediated cytotoxicity (Figure S3).

To determine whether inhibition of the classic cAMP-PKA signaling pathway can also protect ECs from EBOV infection, HUVECs were pretreated with 10 µM H89 [47]—a specific inhibitor of PKA—and infected with EBOV. After 72 h p.i., no protective effect was observed in H89-treated cells compared to the Vehicle-treated group (Figure 2A,B). These results indicate that EPAC1 plays an important role during EBOV infection in ECs and affects viral infection independently of the classic PKA signaling pathway.

ESI09-mediated efficacy in other experimental EC models was also evaluated. Similar experiments using hCMECs and BMECs from WT mice were performed. As shown in Figure S4A,B, reduced EBOV infection was observed also in these ESI09-pretreated ECs (*p* < 0.005).

In order to rule out that the ESI09-mediated effect is limited to EBOV, similar experiments were performed with MARV (see Supplemental Materials for GenBank accession number), another highly virulent member of the family Filoviridae. It was found that, compared to the Vehicle group, ESI09 treatment in HUVECs infected with MARV also attenuated formation of VAPF (Figure S4D) and reduced the number of viral RNA copies in media at 72 h p.i. (Figure S4C) (*p* < 0.001).

### 3.3. ESIs Protect ECs When Given 24 h after EBOV Infection

To assess whether postexposure protection of ECs against EBOV infection can be conferred by pharmacological inactivation of EPAC1, HUVECs were treated with ESI09 and NY0123 at 24 h after infection with EBOV at an MOI of 0.5. At 72 h p.i., decreased viral load in cells treated with ESI09 (*p* < 0.05) or NY0123 (*p* < 0.01) (Figure 2O) and cell culture medium from cells treated with ESI09 (*p* < 0.05) or NY0123 (*p* < 0.01) (Figure 2P) were observed compared with Vehicle-treated groups. Similar inhibitory effects were confirmed by observing a decrease in the formation of VAPF in cell monolayers post-treated with ESI09 or NY0123 (*p* < 0.05) (Figure 2Q). These data support that pharmacological inactivation of EPAC1 imparts a postexposure effect against EBOV infection in ECs.

### 3.4. ESI09 Blocks the Early Stage of EGP-VSV Infection in ECs

**Figure 3:**
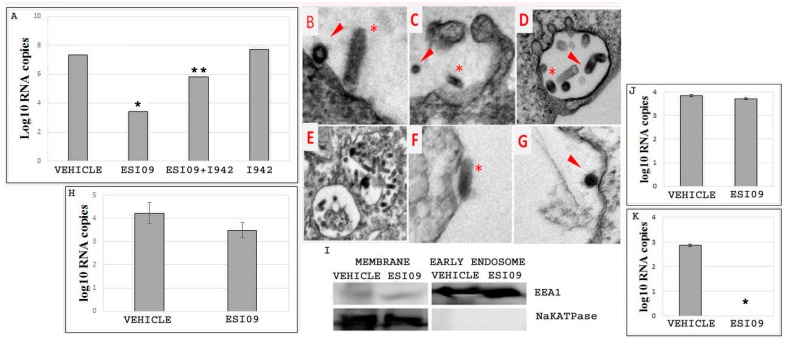
ESI09 blocks the early stage of vesicular stomatitis virus expressing EBOV glycoprotein (EGP-VSV) infection in endothelial cells. (**A**) The number of EGP-VSV viral RNA copies detected at 24 h p.i. in the Vehicle-, ESI09-, ESI09+ I942-, and I942-pretreated HUVECs. *N* = 3 for each group. * *p* < 0.05 compared to the Vehicle group, ** *p* < 0.05 compared to the ESI09 group. (**B**–**G**) EM detection of EGP-VSV particles in HUVECs pretreated with DMSO (**B**–**E**) and ESI09 (**F**–**G**) at 6 h p.i. with EGP-VSV (at an MOI of 200). Arrows highlight EGP-VSV particles in cross-sections, and asterisks indicate particles in longitudinal sections. Scale bar, **B**–**G**, 2 µM. (**H**) The number of viral RNA copies detected in the membrane-bound fractions from the binding assay of Vehicle- and ESI09-pretreated HUVECs at 15 min p.i. with EGP-VSV at an MOI of 20. *N* = 3 for each group. (**I**) Confirmation of the qualities of the samples from the membrane and early endosome fractionations. (**J**) The number of EGP-VSV viral RNA copies detected at 1 h p.i. at an MOI of 200 in the membrane fractions of Vehicle- and ESI09-pretreated HUVECs. *N* = 3 for each group. (**K**) The number of EGP-VSV viral RNA copies detected at 1 h p.i. at an MOI of 200 in the early endosome fractions of Vehicle- and ESI09-pretreated HUVECs. *N* = 3 for each group. * *p* < 0.001 compared to the Vehicle group.

To further dissect the cellular mechanisms by which EPAC1 is involved in filoviral infection, EGP-VSV, which mimics EBOV infection, was employed. First, experiments were performed using the same treatment conditions as described for EBOV previously. HUVECs were pretreated with 5 µM ESI09 or DMSO, then infected with EGP-VSV at an MOI of 1 and incubated for 24 h. The efficacy of ESI09 was evaluated by qPCR. As shown in Figure 3A, pretreatment with ESI09 significantly protected cells against EGP-VSV infection (*p* < 0.05), whereas Vehicle-treated cells remained highly susceptible to infection. I942 [43] (5 µM) pretreatment only did not increase infectivity significantly. Interestingly, however, treatment with a combination of ESI09 (5 µM) and I942 [43] (5 µM) before challenge with EGP-VSV restored viral infection in HUVECs (*p* < 0.05) (Figure 3A).

To identify which stage of the EBOV life cycle was affected by inactivation of EPAC1, ESI09- or DMSO-pretreated HUVECs were inoculated with EGP-VSV at an MOI of 200 for 6 h and examined by EM (Figure 3B–G). In HUVECs pretreated with DMSO, we observed that EGP-VSV triggered cell membrane ruffling and entered cells mostly by a macropinocytosis-like pathway [45,48]. EM studies also showed single or agglomerated EGP-VSV particles in the vesicular compartments (Figure 3B–E). In contrast, in HUVECs pretreated with ESI09, EGP-VSV particles were observed on the surfaces of cell membranes (Figure 3F,G). EGP-VSV particles were rarely observed during the macropinocytosis-like process, and few EGP-VSV particles were found in vesicular compartments. These data suggest that inactivation of EPAC1 affects the early stages of viral infection, rather than later fusion or replication stages.

To examine whether ESI09 affects viral binding to ECs, a virus binding assay was performed by incubating DMSO- or ESI09-pretreated HUVECs with EGP-VSV at an MOI of 20 for 15 min to allow the viral particles to bind to the cell surfaces. Bound viruses were analyzed by qPCR. As shown in Figure 3H, the number of viral RNA copies of membrane-bound viral particles detected in the ESI09-pretreated group was not significantly different when compared to the Vehicle-pretreated group. This result suggests that inactivation of EPAC1 has no significant effect on EBOV binding to the EC surface.

To better define the inhibitory effect of ESI09 on EBOV infection, we measured the number of EGP-VSV RNA copies present in the membrane and early endosome fractions. HUVECs grown in flasks pretreated with ESI09 or DMSO were incubated with EGP-VSV at an MOI of 200 for 1 h, then rinsed thoroughly before being processed for membrane and early endosome fractionation, respectively (Figure 3I). In the membrane fractions, viral RNA was detected in both groups and revealed no significant differences (Figure 3J), whereas no viral RNA was detected in early endosome fractions from the ESI09-treated group. As expected, viral RNA was found in the early endosome fractions of the Vehicle-treated group (*p* < 0.001) (Figure 3K). These results confirm that no viral RNA copies are detected in early endosome compartments of ECs following inactivation of EPAC1.

Taken together, these data suggest that ESI09 affects the early stage of EBOV infection that occurs after initial viral binding to the cell surface, but before the formation of the early endosome that includes viruses.

### 3.5. ESI09 Regulates EBOV Entry in a PI3K-Dependent Manner

**Figure 4:**
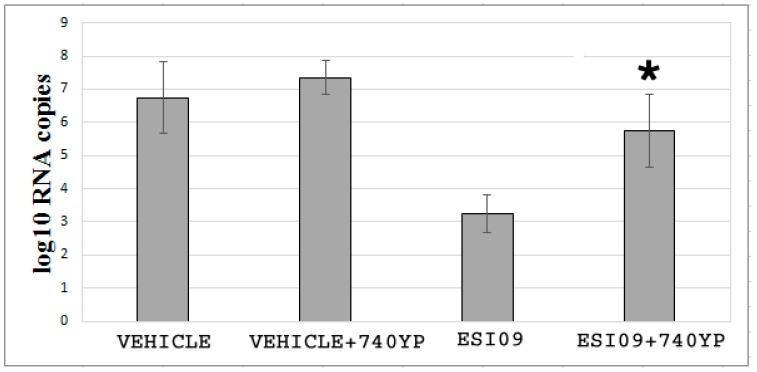
ESI09 regulates EBOV entry in a PI3K-dependent manner. qPCR assessment of the effects of PI3K on EPAC1-mediated EGP-VSV infection. The number of viral RNA copies detected at 1 h p.i. with EGP-VSV at an MOI of 200 in Vehicle-, Vehicle+740YP-, ESI09-, and ESI09+740YP-pretreated HUVECs. *N* = 3 for each group. * *p* < 0.05 compared to the ESI09 group.

Earlier studies have shown that PI3K—a family of related intracellular signal transducer enzymes capable of phosphorylating the 3-position hydroxyl group of the inositol ring of phosphatidylinositol—is critical for the entry of EBOV [49,50]. ESI09 has been documented to suppress PI3K-dependent Akt phosphorylation [51,52]. We confirmed this in our EC model (Figure S5). To further evaluate the effects of PI3K on the infectivity after inactivation of EPAC1, we incubated ESI09-pretreated HUVECs with the PI3K-specific activator 740YP (10 µM) [53] for 3 h before challenging with EGP-VSV. Activation of PI3K in ESI09-pretreated ECs significantly reduced the protective efficacy of ESI09 compared with the ESI09-treated group that did not receive 740YP (Figure 4) (*p* < 0.05), as determined by qPCR assessment of viral RNA copy numbers. The 740YP treatment did not significantly affect the viral load in the Vehicle-pretreated group.

Taken together, these data suggest that regulation of EPAC1 on EBOV entry may require the involvement of the PI3K signaling pathway(s).

## 4. Discussion

The majority of enveloped viruses depend on endocytosis for entry [54,55]. The viral glycoprotein (GP)-mediated viral uptake by endocytosis and membrane fusion provide opportunities for exploiting potential druggable target(s) to control filovirus entry into the cell [56,57,58]. Significant insights have been gained from intensive studies into the mechanism(s) underlying the membrane fusion between filovirus and endocytic vesicles that introduces the virus genome into the cytoplasm, where the subsequent steps of the viral replication cycle are carried out. However, earlier events during EBOV uptake by endocytosis, before the virus fully enters the cell, remain enigmatic. ECs play an important role in the pathogenesis of a lethal filoviral infection. Using ex vivo and in vitro models, we have demonstrated that deletion of the *EPAC1* gene or inactivation of EPAC1 by ESIs at nontoxic concentrations significantly attenuates EBOV infection in both vasculature and ECs, while activation of EPAC1 using ESA restored EBOV infection in ESI09-pretreated ECs. By combining distinct and independent approaches, we found that inactivation of EPAC1 blocks filoviral uptake. Our experiments presented here suggest that PI3K pathway-mediated filoviral endocytosis is the target of the pharmacological manipulation of the cAMP-EPAC system. Activation of PI3K in ESI09-treated ECs significantly reduced the protective efficacy of ESI09 but did not significantly affect viral infectivity in Vehicle-treated groups. This observation suggests that EPAC1 governs EBOV uptake into ECs. The present report is the first study to reveal the involvement of the cAMP-EPAC signaling pathway in the entry of enveloped viruses. Importantly, the cAMP-EPAC signaling axis can be exploited as a potential target for the development of compounds to control filoviral infections.

The finding that the intracellular cAMP receptor EPAC is important for EBOV infection in ECs is consistent with previous reports regarding the roles of EPAC in intracellular pathogen infections [40,41]. One study recently demonstrated that MERS-CoV and SARS-CoV require EPAC1 for viral replication. Pretreatment with ESI09 exerts antiviral effects in epithelial cell lines infected with MERS-CoV [40]. We have previously reported that deletion of the *Epac1* gene protects mice from a lethal dose of spotted fever group rickettsiae [41], which are obligate intracellular bacteria. In that study, inhibition of EPAC1 suppressed bacterial adhesion and invasion. Taken together, these data provide a strong link between the host cAMP-EPAC signaling axis and intracellular pathogen infections.

A previous study using a cAMP-PKA-specific inhibitor demonstrated that hepatitis C virus exploits the cAMP-PKA signaling system to facilitate its entry and infectivity [31]. To determine whether this classic cAMP signaling is critically involved in EBOV infection of ECs, HUVECs were pretreated with a cAMP-PKA-specific inhibitor before infection with EBOV, and no protective effect was observed. These results confirm that EPAC1 plays an important role during EC EBOV infection and affects viral infection independently of the classic cAMP-PKA signaling pathway.

The initial encounter between EBOV and EC occurs at the plasma membrane. EBOV must adhere to the cell surface to trigger the host’s endocytic plasma membrane dynamics. During binding to the host surface, EBOV virions interact with various cell membrane molecules, including nonspecific receptors. Two types of nonspecific receptors have been identified: C-type lectins [59] and phosphatidylserine receptors [48,54,60,61]. In the present study, the viral binding assay using EGP-VSV showed no significant differences between DMSO- and ESI09-treated HUVECs, suggesting that EBOV binds to the endothelial surface in an EPAC1-independent manner. However, ultrastructural experiments revealed that EGP-VSV is internalized into ECs via macropinocytosis in an EPAC1-dependent manner. These findings are compatible with previous studies that showed that the cAMP-EPAC signaling pathway is required for the regulation of compartmentalization of endosomes [62,63,64]. Importantly, inactivation of EPAC1 by ESI09 dramatically reduced the number of viral RNA copies in early endosome compartments, suggesting that EPAC1 is a potential target for controlling EBOV infection in ECs using ESIs.

Owing to the generally large size of filamentous particles, these viruses are predominantly taken up through macropinocytosis [48,50,65,66], in which classic clathrin-, caveolin-, and phagocytosis-mediated endocytosis is not critically involved [50,65]. Our finding from EM studies demonstrated that inactivation of EPAC1 abrogated the creation of EGP-VSV-induced macropinocytosis formed in the ruffling regions of the endothelial plasma membrane, suggesting that EBOV GP-mediated virus uptake requires the involvement of the host cAMP-EPAC1 signaling pathway.

Many intracellular pathogens exploit host cell signaling pathways to facilitate their entry [28,29,30,31,32]. However, deeper insight into the requirement of cell signaling pathways for EBOV uptake remains scarce. One of the current views of EBOV uptake is that cholesterol-rich lipid rafts in the host plasma membrane serve as an important platform used by EBOV to enter cells [67]. Although it is the least abundant phospholipid in the cell membrane, phosphatidylinositol is one of the most versatile signaling molecules in cells and plays an important role in endocytosis [68]. PI3K plays a well-characterized role in macropinocytosis, where it orchestrates signaling and cytoskeletal modulation during protrusion, extension, and closure of macropinosome formation [68]. Activation of PI3K has been observed upon virus binding in a number of infection models of enveloped viruses [68]. EBOV induces its internalization into host cells by a viral GP-triggered entry mechanism employing the PI3K-Akt signaling pathway [49,50]. Considering that activation of EPAC stimulates the PI3K-Akt signaling pathway [51], it is conceivable that EPAC1 may regulate EBOV uptake by modulating the cellular PI3K-Akt signaling system. EPAC1 is known to regulate all of the aforementioned cellular functions, but the precise molecular mechanism(s) by which EPAC1 controls EBOV entry is not clear. Further research into understanding the signal cross-talk between EPAC1 and PI3K signaling pathways hijacked by EBOV during macropinocytosis is currently ongoing.

We observed postexposure protection of ECs against EBOV infection that was conferred by pharmacological inactivation of EPAC1, but the biological basis at the cellular level remains unclear. Using an ex vivo model, we detected sporadic EBOV viral antigen signaling colocalized with an EC-specific marker, vWF, along the tunica intima of the aortic rings at 72 h p.i. (Figure 1A). Using IF microscopy to measure the number of VAPF in the EBOV-infected cell monolayer at 72 h p.i., viral antigen-positive ECs were detected in cell clusters. No viral antigens were visualized in the cells surrounding VAPFs. It appears that EBOV replicates in ECs in clusters rather than in neighboring cells in the same monolayer, suggesting that there is a window following the initial infection of the ECs in the cluster to protect neighboring cells from viral infection. However, future observation of longer periods of time postinfection will provide more accurate information to support the potential postexposure protection conferred by ESIs. Further in vivo examinations should provide additional data to support the hypothesis that ESIs could be used to develop a therapeutic strategy against filoviral infection.

Despite significant progress in the development of cAMP analogues as EPAC agonists [69], the EPAC isoform selectivity of these cAMP analogues and their potential off-target effects on other molecules, such as cAMP phosphodiesterases, remain challenging [70,71]. Technically, since cAMP analogues are bioactivated by esterases, cells should be cultured without sera (see http://www.biolog.de/media/TechInfo/C%20051.pdf), which restricts applications using primary endothelial cells. A family of novel non-cAMP EPAC1 agonists have been developed that represent the first-in-class isoform-selective EPAC1 activator; these compounds have the potential to suppress proinflammatory cytokine signaling, thereby reducing the risk of side effects associated with general cAMP-elevating agents that activate multiple response pathways [43]. One such agonist, ESA I942, was employed in our study. It displayed a capability to exert its pharmacological effects in complete EC culture medium, and induced enhanced EGP-VSV infection in HUVECs, thereby supporting the important role that EPAC1 plays during EBOV infection.

Nevertheless, there were limitations in our present study to elucidate the exact mechanism(s) underlying the inactivation of EPAC1 attenuating EBOV infection in ECs. First, the ex vivo model allows us to perform comparative studies between WT and KO groups. However, it cannot mimic the complicated host responses in the context of in vivo infection. Second, a non-mouse-adapted Ebola virus used in our ex vivo mouse model may not be directly translated into in vivo applications since wild-type virus is not pathogenic in mice. Third, histological requirements, including the appropriate orientation of the aortic ring during embedding to show the intima layer during microtomy and effective antigen retrieve during IF staining, make it difficult to optimize EC-specific staining of vWF. Last, measuring the level of Akt-1 phosphorylation between Vehicle-, ESI09-, and I942-treated groups only provides indirect evidence to support the hypothesis that the PI3K signaling system is involved in the mechanism underlying the protection against EBOV infection via inactivation of EPAC1. An accurate mechanism is yet to be elucidated.

In conclusion, our studies combined in vitro primary human endothelial systems with an ex vivo model to reveal a novel role for EPAC1 in EBOV infection. In vascular ECs, EPAC1 governs EBOV internalization via regulation of PI3K pathway-mediated filoviral macropinocytosis. Deeper insights will be gained by future in vivo studies using *EPAC1*-null mouse models of EBOV infection and in vivo efficacy testing of ESIs using the wild-type mouse model of filoviral infections. These future studies should hopefully pave the way toward the development of novel, broad-spectrum antiviral therapeutics to combat filoviral infections.

## Supplementary Materials

The following are available online at http://www.mdpi.com/1999-4915/10/10/563/s1, Figure S1: Representative immunostaining of EBOV viral antigens in viral antigen-positive foci. Figure S2: Higher magnification images of the inserts in Figure 1A. Figure S3: Cytotoxicity of DMSO, ESI09, NY0123, and I942 in HUVECs. Figure S4: Pharmacological inactivation of EPAC1 protects different ECs from EBOV or MARV infection. Figure S5: Effect of pharmacological manipulation of EPAC1 on Akt-1 phosphorylation in ECs.
