# Supplementary material for: Exchange Protein Directly Activated by cAMP Modulates Ebola Virus Uptake into Vascular Endothelial Cells
Source: Viruses. 2018 Oct 16;10(10):563. doi: 10.3390/v10100563 (PMC6213290; doi:10.3390/v10100563)
Supplement: Supplementary file 1 [file viruses-10-00563-s001.pdf]

Supplemental Materials

Materials and Methods

Antibodies and Reagents

A hyperimmune polyclonal anti-Ebola virus (EBOV) antibody was produced by immunizing a rabbit with Zaire EBOV (strain Mayinga); the virus was grown in RK13 cells (Central Diseases Control, Dr. Thomas Ksiazek). Rabbit anti-von Willebrand factor (vWF) polyclonal antibody and rabbit anti-early endosome antigen 1 (EEA1) monoclonal antibody were purchased from Invitrogen (Carlsbad, CA). Rabbit anti-Akt polyclonal antibody and rabbit phospho-Akt (Ser473) polyclonal antibody were purchased from Cell Signaling Technology (Danvers, MA). Mouse anti-human Na+, K+ ATPase antibody was purchased from BD Biosciences (San Jose, CA). Unless otherwise indicated, all reagents were purchased from Thermo Fisher Scientific (Rockford, IL).

Cell Culture and Virus

Human umbilical vein endothelial cells (HUVECs) (Cell Applications, Atlanta, GA) or human cerebral microvascular endothelial cells (hCMECs) (Applied Biological Materials, Richmond, BC, Canada) were grown in Endothelial Cell Growth Medium (Cell Applications, Atlanta, GA) supplemented with 10% heat-inactivated fetal bovine serum (FBS) with humidity in 5% CO_2 at 37 °C. The media was changed every 48 h. Cells were allowed to grow for up to 48 h until 90% confluence of the monolayer was achieved. Cells were maintained in Endothelial Cell Growth Medium during the experiments. Cell passages 3–6 were used for all experiments. African green monkey kidney epithelial Vero E6 cells (ATCC CRL-1586) were grown in Dulbecco's modified Eagle's medium (DMEM) with 5% FBS with humidity in 5% CO_2 at 37 °C. Mouse primary brain microvascular endothelial cells (BMECs) were isolated from brain tissues of pathogen-free laboratory 6–8-week-old C57BL/6 wild-type and *Epac1*-null mice using a published protocol [1]. BMECs were grown in Endothelial Cell Growth Medium (Cell Applications) supplemented with 10% FBS with humidity in 5% CO_2 at 37 °C. Cell passages 2–3 were used for all experiments.

EBOV (199510621 Zaire strain, GenBank accession number KR824526) and Marburg virus (200501379 Angola strain, GenBank accession number KU978782) stocks were propagated in Vero E6 cells in Earl's modified Eagle's medium (EMEM) with 2% FBS at 37 °C. The freeze-thaw technique was used for virus harvesting. EBOV titers were calculated using tissue culture infective dose (TCID50) assays done in a microtiter format using four replicates at 10-fold serial dilutions (10^{-2} to 10^{-8}). After an 11-day incubation period at 37 °C, the cells were fixed with 10% buffered formalin overnight, and then stained with 0.5% crystal violet for 15 min. Virus titers were calculated using the method of Reed and Muench. All work with filoviruses was performed in the biosafety level 4 (BSL-4) facility of the Galveston National Laboratory.

The recombinant vesicular stomatitis virus expressing the glycoprotein (GP) of Zaire EBOV (strain Mayinga) (EGP-VSV) was kindly provided by Drs. Thomas Geisbert and Chad Mire (UTMB). Viral stocks were prepared in Vero E6 cells using DMEM with 2% fetal calf serum, and stored at −80°C. The concentration of EGP-VSV was determined by plaque assay in Vero E6 cell monolayers under a 1.6% Tragacanth gum mixed with 2× DMEM medium (1:1) with 2% FBS overlay. Plaques of EGP-VSV were counted after staining with 0.25% crystal violet in 10% buffered formalin.

Two-Step Real-Time PCR (qPCR)

Total RNA from TriPure Isolation Reagent-diluted supernatants and cell samples was extracted according to the manufacturer's directions and resuspended with 20 μL of diethylpyrocarbonate (DEPC)-treated RNase-free water. RNA was quantified spectrophotometrically at 260 nm using a NanoDrop 2000 Spectrophotometer (Thermo Fisher Scientific). Samples of purified RNA were stored at −80°C. One microgram of each RNA sample was reverse transcribed using the iScript reverse transcription supermix (BioRad, Hercules, CA) with an RNase inhibitor and random hexamer primers according to the manufacturer's instruction. Detection of EBOV was performed using a qPCR TaqMan assay that amplified an 80-bp fragment of the receptor binding and fusion glycoprotein gene fragment of EBOV-Zaire. The primers used were: 5'-TTT TCA ATC CTC AAC CGT AAG GC-3' (forward) and 5'-CAG TCC GGT CCC AGA ATG TG-3' (reverse), and probe FAM-CAT GTG CCG CCC CAT CGC TGC-BHQ1. cDNA samples were run on the Stratagene Mx3000P thermal cycler in a final volume of 20 μL containing 1x TaqMan Fast Advanced Master Mix, 1 μmol of each primer, 0.1 μmol probe, and 100 ng template. The thermal profile for the reaction was: 50 °C for 15 min, 95 °C for 5 min, followed by 45 cycles of 95 °C for 1 s, 60 °C for 20 s, then 1 cycle of 40 °C for 30 s. In each qPCR run, 10-fold serial dilutions of a standard (60,000 EBOV-Zaire Mayinga particles/μL of RNA) were included to confirm the sensitivity of the qPCR method, and noninfected cells (mock) were used as a negative control. A standard curve was used to convert the threshold cycle value (Cq) to the number of RNA copies per milliliter of sample. For Marburg virus (MARV) detection, primers MN FP and MN RP, and probe MN P were used as previously described [2]. Ten-fold serial dilutions of MARV RNA (409,000 MARV-Ravn particles/μl of RNA) were included as a standard and a positive control in each PCR run.

Immunoblotting and Immunoprecipitation (IP)

For immunoblotting assays, equal amounts of soluble protein were subjected to 12% SDS–polyacrylamide gel electrophoresis (SDS-PAGE). Proteins were transferred onto a polyvinylidene difluoride membrane and then incubated with primary antibody at 1:1000 overnight, followed by incubation with a horseradish peroxidase (HRP)-conjugated secondary antibody at 1:2000 for 1 h. Blots were visualized by using an enhanced chemiluminescence kit (LI-COR Biosciences, Lincoln, NE). All experiments were repeated three times.

For the IP study, equal amounts of soluble proteins were mixed with Dynabead Protein G (Invitrogen) conjugated with anti-Akt-1 antibody, and were incubated for 24 h at 4 °C. The Dynabead-antibody-antigen complex pellets were precipitated and separated using DynaMag-2 (Invitrogen). Samples were then separated by gel electrophoresis followed by immunoblotting. All experiments were repeated three times.

Transmission Electron Microscopy (EM)

For EM assays, HUVECs were subjected to fixation, LR White embedding, and ultrathin sectioning as previously described [3]. Uninfected HUVECs were used as mock controls and were subjected to the same procedure.

Supplemental Figure Legends

Supplemental Figure 1: Representative immunostaining of EBOV viral antigens in viral antigen-positive foci. IF staining of EBOV viral antigens in the monolayers of DMSO- or ESI09-pretreated HUVECs at 72 hours p.i. with EBOV at an MOI of 0.5. Scale bars, 100 μm.

Supplemental Figure 2: Higher magnification images of the inserts in Figure 1A. Scale bar, 5 μm.

Supplemental Figure 3: Cytotoxicity of DMSO, ESI09, NY123, and I942 in HUVECs. To determine the effects of ESI09, NY123, and I942 on HUVEC viability in vitro, cytotoxicity was determined using the 3-(4,5-dimethylthiazol-2-yl)-5-(3-carboxymethoxyphenyl)-2-(4-sulfophenyl)-2H-tetrazolium (MTS) assay after culturing HUVECs with serial dilutions (1.25, 2.5, 5, 10, and 50 μM) of either ESI09 (**A**), NY123 (**B**), or 5 μM I942 (**A**), respectively, for 24 hours. HUVECs with 10 μM DMSO (**A** and **B**) treatment served as the control. Results are presented as mean ± standard error of the mean of the three independent experiments; each was carried out in triplicate. N = 6 for each group. *p < 0.05 versus control.

Supplemental Figure 4: Pharmacological inactivation of EPAC1 protects different ECs from EBOV or MARV infection. The number of viral antigen-positive foci measured under IF microscopy in the monolayers of human cerebral microvascular endothelial cells (hCMECs) (**A**) and mouse BMECs (**B**) at 72 hours p.i. with EBOV at an MOI of 0.5. N = 30 for each group. *p < 0.005 compared to Vehicle groups. (**C**) The number of viral RNA copies detected in the media from Vehicle- and ESI09-pretreated HUVECs at 72 hours p.i. with MARV at an MOI of 0.5. N = 30 for each group. *p < 0.001 compared to the Vehicle group. (**D**) The number of viral antigen-positive foci measured under IF microscopy in the monolayers of HUVECs at 72 hours p.i. with MARV at an MOI of 0.5. N = 30 for each group. *p < 0.001 compared to the Vehicle group.

Supplemental Figure 5: Effect of pharmacological manipulation of EPAC1 on Akt-1 phosphorylation in ECs. Representative levels of p-Akt-1 (S473) in HUVEC cells pretreated with Vehicle, ESI09, or I942.

References

1. Ruck T, Bittner S, Epping L, Herrmann AM, Meuth SG. 2014. Isolation of Primary Murine Brain Microvascular Endothelial Cells. J Vis Exp 93: 52204.

2. Weidmann M, Mühlberger E, Hufert FT. 2004. Rapid detection protocol for filoviruses. J Clin Virol 30:94-9.

3. Popov VL, Yu X, Walker DH. 2000. The 120 kDa outer membrane protein of Ehrlichia chaffeensis: preferential expression on dense-core cells and gene expression in Escherichia coli associated with attachment and entry. Microb Pathog 28:71-80.

1.

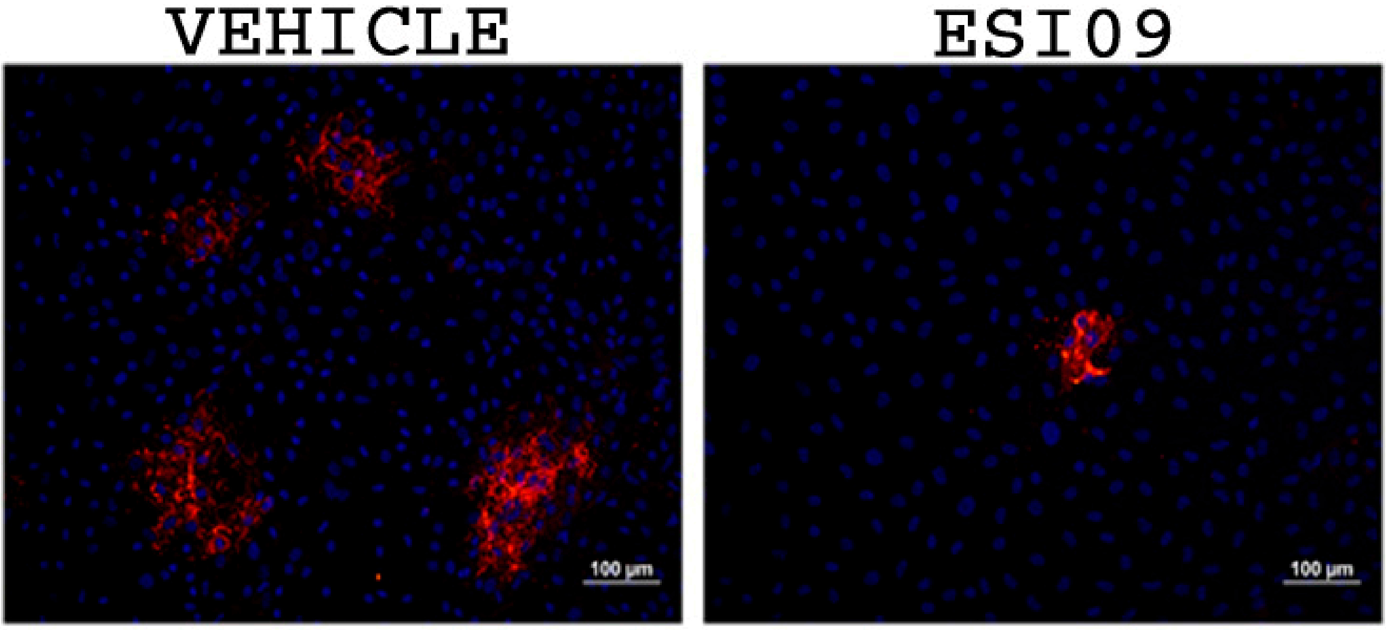

Figure S1. Representative immunostaining of EBOV viral antigens in viral antigen-positive foci.

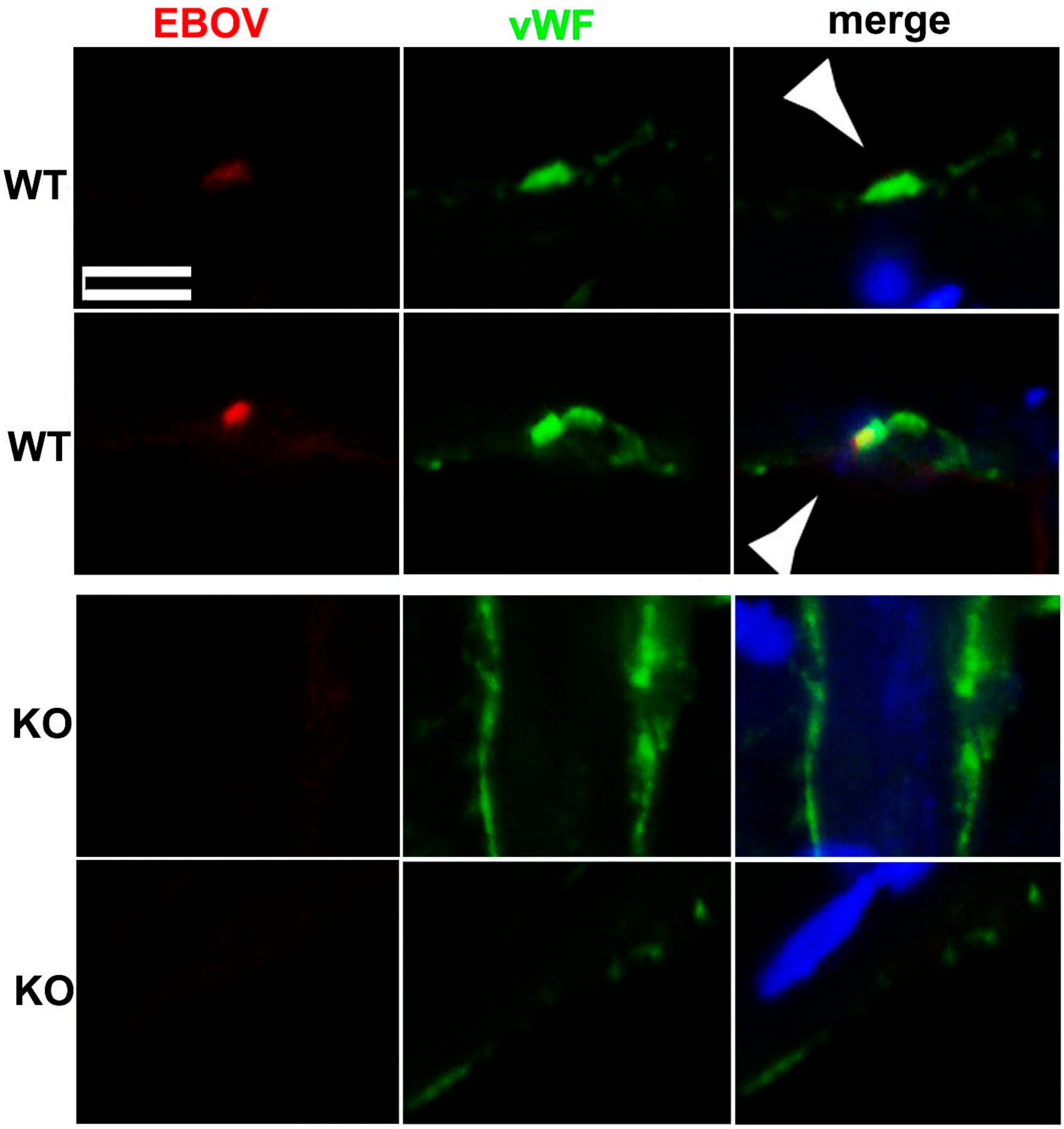

Figure S2. Higher magnification images of the inserts in Figure 1A.

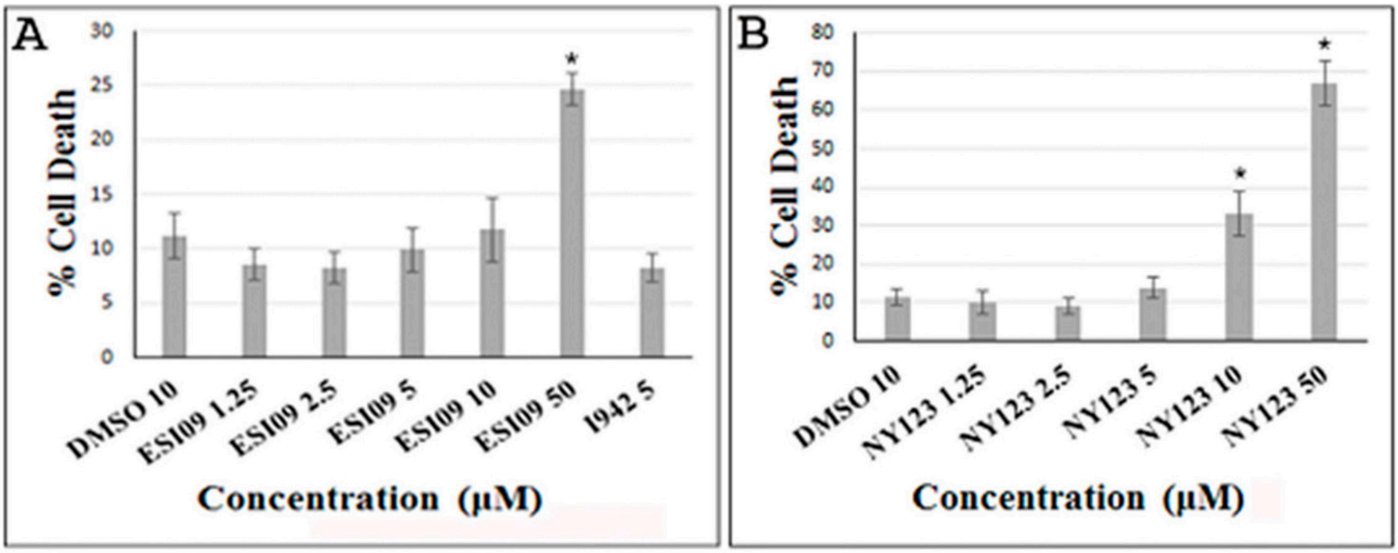

Figure S3. Cytotoxicity of DMSO, ESI09, NY123, and I942 in HUVECs.

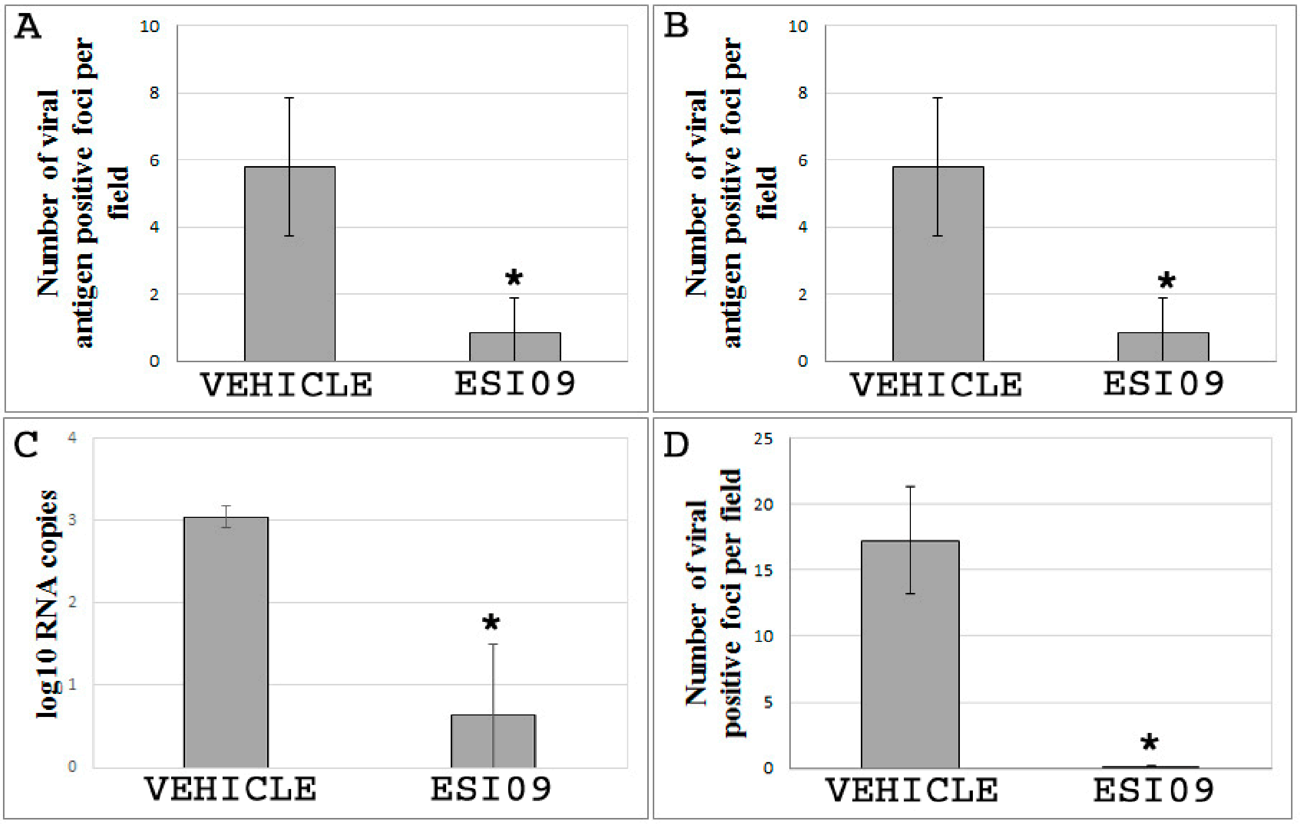

Figure S4. Pharmacological inactivation of EPAC1 protects different ECs from EBOV or MARV infection.

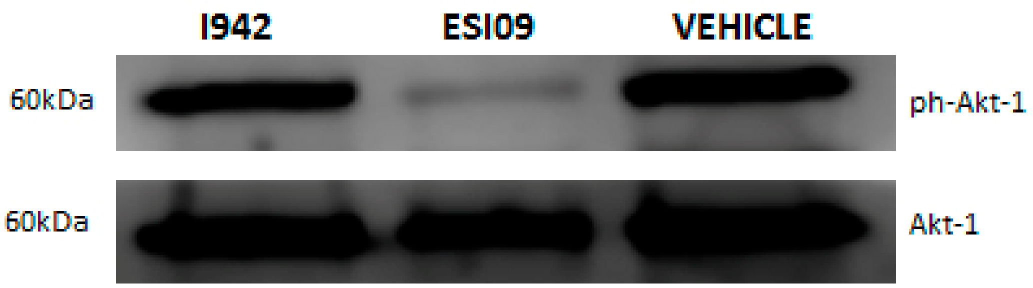

Figure S5. Effect of pharmacological manipulation of Epac1 on Akt-1 phosphorylation in ECs.
